# Impact of COVID-19 pandemic on the practice of pediatric dentistry in the United Arab Emirates: a cross-sectional survey

**DOI:** 10.1186/s43054-022-00121-2

**Published:** 2022-11-07

**Authors:** Hisham Yehia El Batawi, Zain Sawalha, Mai Almomani

**Affiliations:** 1 University of Sharjah, Sharjah, United Arab Emirates; 2 Gulf Medical University, Ajman, United Arab Emirates

**Keywords:** Pediatric, Dentistry, COVID, Corona virus epidemic infection control

## Abstract

**Background:**

The aim of this study was to assess the impact of COVID-19 epidemic circumstances on the practice of pediatric dentistry in the United Arab Emirates.

**Methods:**

An online questionnaire was distributed among members of Emirates Club of Pediatric Dentistry. The questionnaire was divided into three sections: (1) dentists’ demographic characteristics, (2) general knowledge and attitude toward COVID-19, (3) practice of pediatric dentistry during COVID-19 pandemic.

**Results:**

Female pediatric dentists were double the males and the age distribution tended towards the younger age group. Most of the participants had a satisfactory level of knowledge about COVID-19 and 100% of them obtained this knowledge from the health authorities. Around one third of the participants were not confident of their ability to work safely during the epidemic. There was a marked diversity between pediatric dentists regarding the priority services to be delivered to children during the COVID-19 peak.

**Conclusion:**

A majority of pediatric dentists in the UAE are well informed regarding COVID-19 and its prevention. The economic impact of the epidemic has disrupted pediatric dentists’ willingness to prioritize certain dental services over others. This is related to safety concerns, financial urge, and patient needs.

## Background

Based on its mode of transmission and high virulence, COVID-19 was declared in January [1] as a public health emergency by the World Health Organization (WHO). COVID-19 high virulence involves direct human contagiousness by physical proximity and subsequent contact with saliva and respiratory fluids.

Recent studies have demonstrated the role of the oral mucosa in COVID-19 infection, in addition to expressing the ACE2 receptor in salivary glands of asymptomatic patients, thus being one main possible source of transmission. In addition, contaminated surfaces in the dental office perpetuate the virus’ survival for up to 5 days [2–5]. 

In 2020, [6], Lee et al. reported that COVID-19 infections among children are uncommon. Among the 44,672 confirmed cases in china of as of 11th of February 2020, 416 (0.9%) were aged 0–10 years and 549 (1.2%) aged 10–19 years. In 2020, [7], Gaffney et al. reported increased risk of COVID-19 complications among teachers and care providers living with low socio-economic school-aged children in USA. Accordingly, these findings may cast concerns on the practice of pediatric dentistry due to its nature that involves physical proximity between the dentist and child, with unavoidable generation of aerosols contaminated with saliva, blood droplets, and pathogens [8].

COVID-19 guidelines for dentists were published and updated regularly via the American Dental Association (ADA), The World Health Organization (WHO), the Center for Disease Control and prevention (CDC) and the American Academy of Pediatric Dentistry (AAPD) as reviewed by Al Halabi et al. in [9]. Despite these efforts, it was reported in [10] by Sarfaraz et al. that the levels of knowledge among dentists regarding infection control and use of personal protection equipment were unsatisfactory.

In the UAE, COVID-19 was first detected in early March 2020. In January 2021, more than 300,000 cases were reported among which 1125 reported deaths. Although UAE a central hub of cultural and ethnic diversity, these numbers were much lower than expected due early implementation of governmental efforts in containing the epidemic via lockdown and restriction of social gathering regulations. Among these regulations were the restriction of dental services to address only emergency patient dental needs [11].

Despite the potential risk of transmission in the dental operatory, the studies assessing the risk of COVID-19 transmission during dental care are few not to mention those studies assessing its impact on the delivery of pediatric dental services [12].

The aim of this work is to study the impact of COVID-19 circumstances on the practice of pediatric dentistry in UAE. The study is an attempt to assess pediatric dentists’ knowledge, attitude, and preparedness for the future should another epidemic takes place once more.

## Methods

A cross-sectional survey of pediatric dentists in the United Arab Emirates was conducted from 1st February 2022 till 30th March 2022 using a questionnaire targeting their knowledge, perception, and attitude towards the practice of pediatric dentistry in COVID-19 circumstances. The study population consisted of 192 registered members of the Emirates Pediatric Dentistry Club (EPDC) who exclusively practice pediatric dentistry in UAE. Most of pediatric dentists in UAE enjoy the membership of this organization. Therefore, targeting the club’s members would serve the aim of the study. University of Sharjah research ethics committee was informed on the procedures and nature of this survey prior to its implementation.

### Structure of the questionnaire

The questionnaire was prepared in English language after reviewing relevant literature and published guidelines provided by the American Dental Association (ADA) and the American Academy of Pediatric Dentistry. The questionnaire is based on questionnaires used by [13, 14] with modifications.

### Sample size

Initially, we targeted 192 registered members of the Emirates Pediatric Dentistry Club (EPDC) who exclusively practice pediatric dentistry in UAE. Initially, 72 pediatric dentists responded in addition to 31 responded upon additional reminding mail which was sent after 2 weeks of the initial mail. Therefore, the total number of contributing pediatric dentists included in the study was 103

A questionnaire draft was first prepared and examined by three pediatric dentists and was modified to ensure validity and applicability of the final version to UAE work environment.

The final questionnaire used three types of closed format questions namely, Importance, Likert, and Dichotomous including 20 mandatory questions divided into five sections:

1. Introductory part including three questions on demographics (age, gender) and nature of practice.
2. Section two of four questions targeting knowledge of practicing pediatric dentists on COVID-19 pandemic, clinical picture, and modes of infection.
3. Section three; composed of seven questions targeting on the different attitudes of pediatric dentists towards COVID-19 and their willingness to continue practicing during COVID-19 circumstances.
4. Section four questions on preferred protective measures and policies performed by pediatric dentists during the COVID-19 pandemic and lockdowns.
5. Section five of two questions on which dental services do pediatric dentists regard as safe or conductive to the least possible risk of infection during COVID-19 pandemic.

The consent form and questionnaire were distributed through an email list of all members of the Emirates Pediatric Dentistry Club (EPDC). Anonymity was ensured, tracing the identity of contributing pediatric dentists was impossible.

The questionnaire was formulated on “Google Forms” format and the link was distributed on email. One reminder Email was sent 2 weeks after the first announcement. On the settings of the questionnaire, responses were set to “1” to avoid duplicate responses from a single respondent.

Descriptive statistics were used to summarize and analyze the data using SPSS software by IBM corporation.

## Results

The survey was conducted on 192 members of the EPDC. Initially, 72 pediatric dentists responded in addition to 31 responded upon additional reminding mail. Therefore, the total number of contributing pediatric dentists included in the study was 103 out of 192 (53.65%).

Practicing pediatric dentists in UAE were found to be 42% below the age of 30, the percentage decreased by age (Table 1). Our data also showed that the almost two thirds (66.9%) of pediatric dentists in the UAE are females.

**Table 1:** Demographics and nature of practice

Item	*N*	%
**Age**		
< 30 years	44	42.7
30–39 years	29	28.1
40–49 years	21	20.3
> 50 years	9	8.7
**Gender**		
Female	69	66.9
Male	34	33
**Nature of practice**		
University	15	14.5
University and private	5	4.8
Private	64	62.1
Private and MOH	0	0
MOH	19	18.4

The nature of practice mostly belonged to the private sector (62.1%) followed by Ministry of Health (MOH, 18.4%) this percentage includes all government run facilities. The least percentage combined both university and private practices. No practicing pediatric dentists were found to combine MOH with private in our study.

### General knowledge on COVID-19 (Table 2)

Our study reported universal agreement on most of the COVID-19 symptoms. All participants recognized fever (100%), headache (100%), and cough (92.2%) followed by sore throat (80.5%), shortness of breath (74.5%), and loss of taste and smell sensation (41.7).

**Table 2:** General knowledge on COVID-19

Item	*N*	%
**COVID-19 signs and symptoms**		
Fever	103	100
Headache	103	100
Cough	95	92.2
Sore throat	83	80.5
Shortness of breath	77	74.5
Loss of smell and taste	43	41.7
**Mode of transmission**		
Hand shakes	48	46.6
Coughing and sneezing	93	90.2
Touching contaminated surfaces	59	57.2
All of the above	99	96.1
**Incubation period**		
2 weeks	84	81.5
1 week	16	15.5
Less than a week	3	2.9
1 day	0	0
**Information source**		
MOH	103	100
Academic institutions, e.g., ADA and AAPD	83	80.5
Friends and family members who got infected	53	51.4
Social media	61	59.2

Concerning knowledge on the modes of transmission, the majority of participants (96.1%) reported coughing, touching contaminated surfaces and hand shaking combined as the modes of transmission. The least percentage (46.6%) reported hand shaking as the main mode of transmitting the disease.

Most participants (81.5%) agreed that the incubation period is within 2 weeks followed by 1 week (15.5%). The least (2.9%) agreed on less than a week as an incubation period while none of the participants reported incubation period less than 24 h.

The main source of information on COVID-19 among participants was the MOH bulletins (100%) followed by Academic institutions like ADA and AAPD (80.5%). Sixty-one participants (59.2%) considered social media as a source of information while 53 (51.4%) considered personal experience with family members and friends who contacted COVID-19.

### Attitude towards COVID-19 (Table 3)

Less than half of the participants (45.6%) in our study perceived COVID-19 as “moderately dangerous” followed by 31% who perceived it as “mildly dangerous”. Almost quarter of participants (23.3%) perceived the disease as “very dangerous” while non-described the COVID-19 risk as over-estimated.

**Table 3:** Pediatric dentists’ attitudes towards COVID-19

	*N*	%
**How do you personally perceive COVID-19?**		
Extremely dangerous	24	23.3
Moderately dangerous	47	45.6
Mildly dangerous	32	31
COVID-19 risk is over estimated	0	0
**Do you consider your practice is well prepared for COVID-19?**		
Yes	32	31
No	43	41.7
Not sure	28	27.1
**Pediatric dentist’s role in educating children and their parents on COVID-19 is….**		
Highly significant	29	28.1
Moderately significant	43	41.7
Mildly significant	28	27.1
Not significant	3	2.9
**Are you aware of the AAPD (or similar organizations) guidelines on COVID-19 infection control?**		
Yes	37	35.9
No	46	44.6
Not sure	20	19.4
**Have you attended a seminar on COVID-19 infection control?**		
Yes	73	70.8
No	4	3.8
Not interested	0	0
Looking forward to	26	25.2
**To what extent do you have confidence in your ability to deliver services safely to a COVID-19-positive child?**		
Greatly confident	36	34.9
Not sure	67	65
**During a high peak of COVID-19 cases, you will**		
Shut down	8	7.7
Open for emergencies	31	30
Open normally	64	62.1

Among the participating pediatric dentists in our study, only (31%) had confidence in their preparedness to manage children with COVID-19 while 41% considered their practice as not prepared to receive children positive for the disease. Those who answered “not sure” constituted 27.1% of the total participants.

Around one third (34.9%) reported confidence in safe delivery of services to children positive for COVID-19 while 65% expressed doubts on their ability to address such patient needs safely.

The percentage of participants who perceived the role of pediatric dentists in educating children and their parents on COVID-19 as “highly significant” was 28.1%. Those who perceived that role as moderately significant were found to be 41.7% while 27.1% perceived it as mildly significant. Only 2.9% perceived the same role as “not significant.”

Less than half of the participants (44.6%) in our study were unaware of AAPD and ADA guidelines on COVID-19. followed by 35.9% who reported well awareness of the same guidelines. Those who knew the presence of these guidelines but did not examine them carefully were found to be 19.4%; their answer was “not sure.”

A majority of participants (70.8%) did attend at least one seminar on COVID-19 followed by 25.2% who are looking forward to attend such an event. The least (3.8%) did not attend any seminars on the subject while none of the participants (0.0%) reported lack of interest.

Our study shows that during a high peak of COVID-19, 62.1% of the participants would open normally followed by 30% who would open only for emergencies. Only 8 participants out of 103 (7.7%) would shut down totally.

### Preferred protective measures and policies during COVID-19 circumstances (Table 4)

The majority of participating pediatric dentists 95.1% agreed on a routine where one parent escorting one child in the operatory. Sixty-six participants (64%) allowed several children with adequate distancing (2 m). Forty-six participants (44.6%) allowed one child at a time with his parents waiting outside. Thirty-two participants (31%) allowed only 1 child and parent appointments with large (1 h) separation intervals.

**Table 4:** preferred protective measures and policies performed by pediatric dentists during the COVID-19 pandemic

	*N*	%
**How do you organize your dental practice with pediatric patients?**		
Child is allowed to come in with only one parent/guardian	98	95.1
Only one child is allowed in while one parent/guardian in waiting room	46	44.6
Several children in waiting room but with adequate distance	66	64
Appointments with considerable time intervals in between	32	31
**The sequence for putting on the protective equipment is**		
Mask, gown, eye protection, gloves	34	33
Gown, mask, eye protection, gloves	56	54.3
Gloves, mask, eye protection, gown	9	8.7
Eye protection, mask, gown, gloves	4	3.8
**Your usual face protection equipment of choice is...**		
FFP1 mask	33	32
FFP2 mask N95	82	79
FPP3 mask	2	1.9
Medical mask	94	91.2
Face shield	98	95.1
**When do you think we can work again without special protection?**		
This year	21	20.3
Next year	74	71.8
Within 2 years	6	5.8
Never again	2	1.9

More than half of our participants followed the “Gown, mask, eye protection, gloves” sequence, followed by 33% who used to wear the mask before the gown. Nine participants (8.7%) reported to start with the gloves followed by mask then eye protection ending with the gown. Four participants (3.8%) used to start with eye protection followed by mask, gown ending with gloves.

The study reported universal agreement on the use of face shields (95.1%) followed by 91.2% using the normal surgical mask. Eighty-two participants reported using N95 masks during COVID-19 circumstances followed by 33 participants (32%) using FFP1 mask. Only 2 participants (1.9%) reported the use of FPP3 mask.

A majority of participants (71.8%) expect that pediatric dentistry would be back to normal without special precautions on next year followed by 20.3% who expect end special precautions this year. Six participants (5.8%) expected ending special protective precautions to end within 2 years. Two participants believed that COVID-19 protective precautions and policies are to stay for good.

### Dental services pediatric dentists would continue to deliver during COVID-19 circumstances (Table 5)

When asked about emergency services suitable for COVID-19 circumstances, temporary fillings were considered by a majority of 98% followed by extractions (90.2%), endodontics for primary teeth (70.8%), long-term restorations (45.6%), sealants and atraumatic restorations (32%), and rotary instruments operative dentistry (19.4). No participants considered prophylaxis nor checkups as an emergency treatment worth delivering during COVID-19 circumstances or lock-down.

**Table 5:** Dental services pediatric dentists would continue to deliver during COVID-19 circumstances

	*N*	%
**If you decide to open your pediatric dental clinic for emergency services only. The services you will deliver are the following:**		
Checkup	0	0
Prophylaxis	0	0
Endodontics for primary teeth	73	70.8
Restorative dentistry using rotary instruments	20	19.4
Sealants, preventive resin restorations (PRR), and atraumatic restorative treatment (ART)	33	32
Temporary fillings	101	98
Long-term restorations	47	45.6
Extractions	93	90.2
**If you decide to open your pediatric dental clinic for limited services. The services you will deliver are the following:**		
Checkup	103	100
Prophylaxis	48	46.6
Endodontics for primary teeth	35	33.9
Restorative dentistry using rotary instruments	24	23.3
Sealants, preventive resin restorations (PRR), and atraumatic restorative treatment (ART).	103	100
Temporary fillings	83	80.5
Long-term restorations	44	42.7
Extractions	86	83.4

When asked should you open for limited scope of service during COVID-19 circumstances, all participants would open for check-up, and sealants (100%) followed by extractions (83.4%), temporary fillings (80.5%), prophylaxis (46.6%), long-term restorations, and endodontics for primary teeth (33.9%). The least percentage was found to be 23.3% for restorative procedures using rotary instruments (burs).

## Discussion

Children make up a small percentage of reported COVID-19 cases, but their role in transmission is still unclear. However, asymptomatic children can have viral loads which in an environment conductive to saliva and blood aerosol like dental environment may have a share in the outbreak.

To our knowledge, this is the second survey among pediatric dentists working in COVID-19 circumstances and the first in the Middle East and North African region (MENA region).

In the current study, the majority of practicing pediatric dentists belonged to the younger age groups. This could be due to the fact that most practicing dentists in UAE are expatriates who may leave their country at a younger age and leave UAE back before getting so old. The same business model also may contribute to the fact that almost two thirds of practicing pediatric dentists in UAE belong to the private sector.

Majority of participating pediatric dentists showed accurate and satisfactory knowledge regarding COVID-19 symptoms as well as its modes of transmission. The majority pf participants also did acknowledge a correct incubation period of 2 weeks as reported by Lee et al, 2020. It is important for practicing dentists to realize that their patients even asymptomatic, would remain potentially infective for 2 weeks after close contact with a COVID-19 patient. These results are close to reports by [13, 14] but differ from earlier reports by [15]. This could be explained on the basis that as time goes by, correct information becomes more abundant.

In the current study, the ministry of health (MOH) was the main source of information, regulations, and recommendations regarding the delivery of dental services during COVID-19 epidemic. This was followed by the American Academy of pediatric dentistry (AAPD) and American Dental Association (ADA). The least percentage (51.1%) reported personal experience through friends and family members as a substantial source of information. During the peak of the epidemic, the UAE MOH, played a significant role in providing education, setting policies and procedures to be followed strictly by health delivery systems including dental facilities during the peak of COVID-19 epidemic.

In this study, all participants considered COVID-19 as dangerous with varying degrees. Almost half of participants (45.6%) regarded COVID-19 as moderately dangerous, around a third (31%) of participants considered it as mildly dangerous while a quarter (23.3%) considered it as very dangerous. This finding is in agreement with the finding reported by [13]. None of our participants regarded COVID-19 risk as over-estimated. This is conflicting with a similar study done on Jordanian dentists by [14] where one third of the participating dentists considered COVID-19 as not a serious public health issue. The conflicting findings could be explained on the fact that their study was early conducted when the full picture of the pandemic was not complete unlike our study which was conducted during a global remission phase of COVID-19.

Almost a third (31%) of our participants were confident that their clinics are well prepared to work safely with COVID-19 children in contrast to 41.7% who expressed lack of preparedness to work safely in COVID-19 circumstances while 27.1% were having doubts. This is more or less is similar to the work done by [13, 16] which was the only research in that context done  on pediatric dentists. This finding reflects the lack of globally unified policies and procedures on the safe delivery of dental services to children during the peak of the epidemic. Also, the majority of practicing pediatric dentists belonged to the private sector which had a limited tolerance towards the financial impact of the epidemic on the practice. Therefore, a substantial percentage of pediatric dentists went on with their practices even though they were not confident on their clinic’s readiness to work safely during the peak of COVID-19.

The financial impact of COVID-19 on the practice of pediatric dentistry in UAE could also explain why 65% of participants were not sure of their personal capabilities to deliver dental services safely to infected children and 62.1% will open normally during a peak of COVID-19.

In the current study, only 28.1% regarded a pediatric dentist’s role in educating the public on COVID-19 as highly significant. This could reflect that COVID-19 pandemic is not originally a dental problem but a medical problem with dental implications. A minority 2.9% answered non-significant for the same question which is a favorable result reflecting that pediatric dentists acknowledge their responsibility towards children and their parents on providing sound facts that might help community efforts combating the epidemic. These results are in agreement with a similar study done by [13] in Austria.

Around one third, 35.9% of respondents were aware of the AAPD and ADA bulletins on COVID-19 in contrast to 44.6% who were not aware with the existence of such guidelines. This reflects the overwhelming availability of information resources to a satisfactory level and that the UAE ministry of health (MOH) provided strict policies and guidelines during the peak of the epidemic. A failure to comply with such guidelines subjected the health service facility to shut down. This could explain why 44.6% in addition to 19.4% overlooked AAPD and ADA published guidelines as they were not obligatory nor impacting the continuity of services during the peak of the epidemic.

A majority of 70.8% attended seminars on the COVID-19 epidemic and 25.2% were looking forward to have the chance to attend such seminars and scientific meetings. This majority could be explained on the existence of a rule by the Emirati MOH that all medical team members should collect a certain number of credit hours of continuous medical education yearly to have their license renewed. A majority of health care workers preferred to have these hours of education to be spent on the topic of COVID-19 infection control. This might explain the difference reported in our study from a similar study by [13] where only 10% of participants attended seminars or workshops on the same topic.

When asked about organizing workflow during COVID-19 circumstances, 64% would allow several children in the waiting area with adequate distance which reflects the financial impact the epidemic might had on the practice of pediatric dentistry. A safer policy of booking child by child appointment with wide time intervals in between would have a negative effect on the income, an outcome which is not well tolerated in a medical community overwhelmed by recruited expatriate dentists hired to generate profit.

It is a common practice among pediatric dentists regardless of the existence of COVID-19 circumstances to allow one child in with only one parent or even ask the parent to wait in the waiting area. This explains the high percentage 95.1% and 44.6% respectively of participants who would apply that policy during COVID-19 epidemic.

Our data shows that 54.3% knew the correct sequence of wearing personal protective equipment (Gown, mask, eye protection, gloves) as stated by Amorim et al in [17] and Beltrán-Aguilar et al in [18]. This was followed by pediatric dentists who start with masks 33%. This reflects the concern of participants over the airborne nature of COVID-19 mode of infection. Moreover, this could also explain the high percentage of participants 95.1% who used face shields regularly as a means of protection against potentially infective droplets of blood and saliva.

It is worth noting that we need to provide additional explanation to our participants regarding the terms used to describe types of masks used by medical personnel. The majority of participants (91.2%) combined the simple surgical mask with face shields followed by 79% who preferred the use of N95 or FFP2 which reflects a positive awareness towards effective use of PPE. This finding is in agreement with other similar study by [16].

At the time of conducting the study, the lockdown measures were starting to ease down. A majority 71.8% are optimistic that next year, pediatric dentistry is going to be practiced as it was before pre COVID-19 era. This is followed by 20.3% who anticipated that pediatric dentistry will be practiced back to normal this year. Only 1.9% expect that COVID-19 has changed the practice of pediatric dentistry for good. These findings are not in agreement with the work by [13] which can be explained on the basis that their study was conducted earlier when COVID-19 global lockdown measures were more tight and the numbers of new cases was on the rise globally.

When asked on types of services participants would provide on emergency basis, the majority (98%) considered temporary fillings to safely arrest the decay process and relief the pain followed by extraction (90.2%) followed by endodontics for primary teeth (70.8%). When the question was rephrased to “what treatments you will choose to provide if you open your office for a limited service?,” checkups and preventive procedures were chosen by 100% of the participants.

The above results may be explained based on some conflicting factors; most of the participants belonged to profit seeking dental facilities with limited tolerance of low income during the epidemic [19]. This financial burden may have urged pediatric dentists to generate profit out of relatively safe services like sealants and Fluoride application. Another driving force might be the sense of duty and responsibility pediatric dentists have urging them to address urgent patient’s needs regardless of the risk of being exposed to potentially infective aerosol of saliva and blood. It is worth noting that during the high peak of COVID-19, all dental rehabilitation for children under general anesthesia was ordered on-hold by the MOH to economize the use of available hospital resources. This sudden stoppage of pediatric dentistry under general anesthesia increased the demand on temporary fillings as a means to postpone the booking for hospital dentistry.

This study has some limitations among which, is that it is a snap shot of a changing situation. For example, the level of knowledge, the severity of the epidemic and the practices of the participants are dynamic and are subject to change as time goes by. To date, this the second study on the impact of COVID-19 on the practice of pediatric dentistry and the first in the Middle East. Pediatric dentists in the UAE are not obliged to join the emirates club of pediatric dentistry. Therefore, the pool from which we collected our data may not be considered as a true representation of all pediatric dentists in the state.

## Conclusion

Within the limitations of the study, pediatric dentists in the United Arab Emirates as a part of the front liners facing the risk of getting infected with COVID-19 had a reliable knowledge enough for the successful and safe delivery of dental services to children in COVID-19 circumstances.

Till present, a unified policy and procedure on the management of dental service delivery to children during an epidemic is lacking and this study recommends the development of such policy.

Health insurance may have a role to play to cover occupational risks and financial disruptions pediatric dentists might encounter should another pandemic breaks out in the future.

## Data Availability

The authors confirm that the data supporting the findings of this study are available within the article [and/or] its supplementary materials.
